# Lessons From the Dot Contraceptive Efficacy Study: Analysis of the Use of Agile Development to Improve Recruitment and Enrollment for mHealth Research

**DOI:** 10.2196/mhealth.9661

**Published:** 2018-04-20

**Authors:** Dominick Shattuck, Liya T Haile, Rebecca G Simmons

**Affiliations:** ^1^ Institute for Reproductive Health at Georgetown University Washington, DC United States; ^2^ Department of Obstetrics & Gynecology University of Utah Salt Lake City, UT United States

**Keywords:** mobile apps, mHealth, higher mobile research, fertility tracker, contraceptive, family planning, fertility awareness method, Dot, contraceptive efficacy

## Abstract

**Background:**

Smartphone apps that provide women with information about their daily fertility status during their menstrual cycles can contribute to the contraceptive method mix. However, if these apps claim to help a user prevent pregnancy, they must undergo similar rigorous research required for other contraceptive methods. Georgetown University’s Institute for Reproductive Health is conducting a prospective longitudinal efficacy trial on Dot (Dynamic Optimal Timing), an algorithm-based fertility app designed to help women prevent pregnancy.

**Objective:**

The aim of this paper was to highlight decision points during the recruitment-enrollment process and the effect of modifications on enrollment numbers and demographics. Recruiting eligible research participants for a contraceptive efficacy study and enrolling an adequate number to statistically assess the effectiveness of Dot is critical. Recruiting and enrolling participants for the Dot study involved making decisions based on research and analytic data, constant process modification, and close monitoring and evaluation of the effect of these modifications.

**Methods:**

Originally, the only option for women to enroll in the study was to do so over the phone with a study representative. On noticing low enrollment numbers, we examined the 7 steps from the time a woman received the recruitment message until she completed enrollment and made modifications accordingly. In modification 1, we added call-back and voicemail procedures to increase the number of completed calls. Modification 2 involved using a chat and instant message (IM) features to facilitate study enrollment. In modification 3, the process was fully automated to allow participants to enroll in the study without the aid of study representatives.

**Results:**

After these modifications were implemented, 719 women were enrolled in the study over a 6-month period. The majority of participants (494/719, 68.7%) were enrolled during modification 3, in which they had the option to enroll via phone, chat, or the fully automated process. Overall, 29.2% (210/719) of the participants were enrolled via a phone call, 19.9% (143/719) via chat/IM, and 50.9% (366/719) directly through the fully automated process. With respect to the demographic profile of our study sample, we found a significant statistical difference in education level across all modifications (*P*<.05) but not in age or race or ethnicity (*P*>.05).

**Conclusions:**

Our findings show that agile and consistent modifications to the recruitment and enrollment process were necessary to yield an appropriate sample size. An automated process resulted in significantly higher enrollment rates than one that required phone interaction with study representatives. Although there were some differences in demographic characteristics of enrollees as the process was modified, in general, our study population is diverse and reflects the overall United States population in terms of race/ethnicity, age, and education. Additional research is proposed to identify how differences in mode of enrollment and demographic characteristics may affect participants’ performance in the study.

**Trial Registration:**

ClinicalTrials.gov NCT02833922; http://clinicaltrials.gov/ct2/show/NCT02833922 (Archived by WebCite at http://www.webcitation.org/6yj5FHrBh)

## Introduction

### Background

Use of smartphone apps for tracking personal health information has grown exponentially in the last decade. Nearly 100,000 such apps are currently available, and almost 1000 enter the market every month [1]. Health care providers, researchers, and app users themselves are concerned about the accuracy of the information provided by these apps, particularly those that claim to give users information on which to base behaviors that affect health outcomes. Building the evidence about which apps provide accurate information and result in the intended behaviors and benefits requires new tech-relevant approaches. Recruiting study participants, particularly for longitudinal mHealth studies, has been challenging; several mHealth studies have adjusted their original strategies to enroll a sufficient number of participants for an appropriate sample size [1,2].

Georgetown University’s Institute for Reproductive Health (IRH) is conducting an efficacy study of the Dynamic Optimal Timing (Dot) app. Dot was developed by Cycle Technologies located in the District of Columbia and is available in app stores for download to iPhones and Android devices. Dot identifies a woman’s fertile window based on her menstrual cycle lengths. The woman enters the first day of her period into the app, and the app provides her with information about her risk of pregnancy each day. She can use this information to achieve or avoid pregnancy. The Dot algorithm adapts its identification of the fertile window to the individual woman as she continues to enter information about her cycle lengths over time [3]. Dot was developed to be suitable means of pregnancy prevention for women with menstrual cycles between 20 and 40 days long and with fewer than 9 days variation in length.

The prospective, longitudinal, contraceptive efficacy study is being supported by the United States Agency for International Development. It is designed to follow the standard guidelines for establishing contraceptive efficacy [4,5], and it takes into account the recommendations for efficacy studies of fertility awareness methods suggested by Trussell and Kost [4]. We adapted the study design to the digital context and integrated participant engagement recommendations from other successful mHealth studies [6,7]. Potential participants were women who had downloaded the app on their Android phones during the recruitment period (February through July 2017) and chosen to use it for pregnancy prevention (rather than achieving pregnancy or tracking their periods). They had entered their most recent period start date at the time of download, and they had continued using the app until they entered a second period start date. There were no literacy criteria specified in the protocol, and Dot currently is only offered in English. In the United States, Android and iPhones hold similar market shares. Outside the United States, Android-based phones far outnumber iPhones. In the United States, we anticipate that a similar percentage of women Android users will download the app as those who downloaded the iPhone app.

The mHealth research literature provided limited guidance regarding recruitment and enrollment for longitudinal studies. The only previous research on an app for pregnancy prevention was a postmarketing study in which all women who downloaded the app were automatically enrolled [8]. But this approach resulted in very high attrition rates, retaining only 3.53% of participants through 13 cycles [8]. It had significant amounts of missing data regarding sexual behavior on fertile days and cycle length. We approached recruitment and enrollment from the perspective of a standard contraceptive efficacy study, setting our goal for recruiting a minimum of 700 women to have at least 255 women complete 13 cycles of use. Thus, we needed women to recognize that they were part of a study, to provide informed consent, to meet specific criteria necessary for a contraceptive efficacy study of this type of method (eg, being sexually active with a male partner, not having used hormonal contraception in the prior 3 cycles, not already being pregnant when they enter the study, aged between 18 and 39 years, and entering their second period start date into the Dot app), and to understand what was being asked of them in terms of data entry.

### Objective

In this paper, we describe the original recruitment and enrollment strategy and the 3 subsequent modifications we made to recruit an adequate sample size. We compare the impact of each change in strategy on the percentage of eligible women who completed the recruitment and enrollment process. In addition to considering the numbers of study participants, we were also concerned about how our sample represents potential users of the Dot app (and other fertility apps). Although we did not design our strategy to ensure that our sample reflected the general US population of women aged 18 to 39 years, we expected that the advertising approach implemented by Cycle Technologies (primarily Facebook and app store advertisements) would reach a fairly representative sample. Facebook was the main advertising platform used to distribute the Dot app, with 2.07 billion active users and 1.37 billion daily users [9]. We examined whether the changes to the recruitment process resulted in shifts in the demographic characteristics of participants.

## Methods

### Dot Study Overview

**Figure 1:**
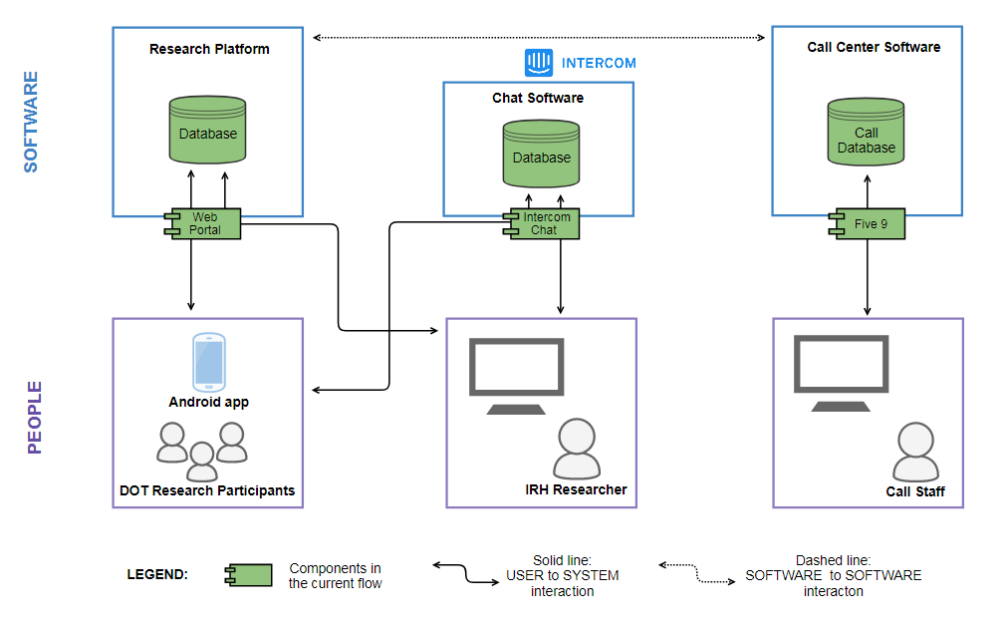
Proofmode’s framework for data collection adapted from Dot study protocol.

For the ongoing prospective, longitudinal, nonrandomized efficacy study of Dot, we recruited and enrolled a cohort of women in the continental United States; in-depth information about the study protocol and approach is available in a previous publication [10]. The study and subsequent modifications were approved by Georgetown University’s Institutional Review Board (IRB), and the study is registered with clinicaltrials.gov (NCT02833922). Participant data over 13 menstrual cycles are collected through the app by activating Proofmode (see Figure 1) at enrollment. Proofmode, developed by the IRH, is the framework for a multicomponent data collection system that overlays the Dot app. This research interface collects study data in real time and allows participants to enter data directly into their phones with or without interacting with a study representative.

Proofmode is divided into 2 components: people and software. Figure 1 is a simplified model of an intricate system that illustrates user-to-system interactions and software-to-software interactions. Data, collected through multiple channels, are stored on a secure research platform that is housed on Georgetown University’s servers [10].

**Figure 2:**
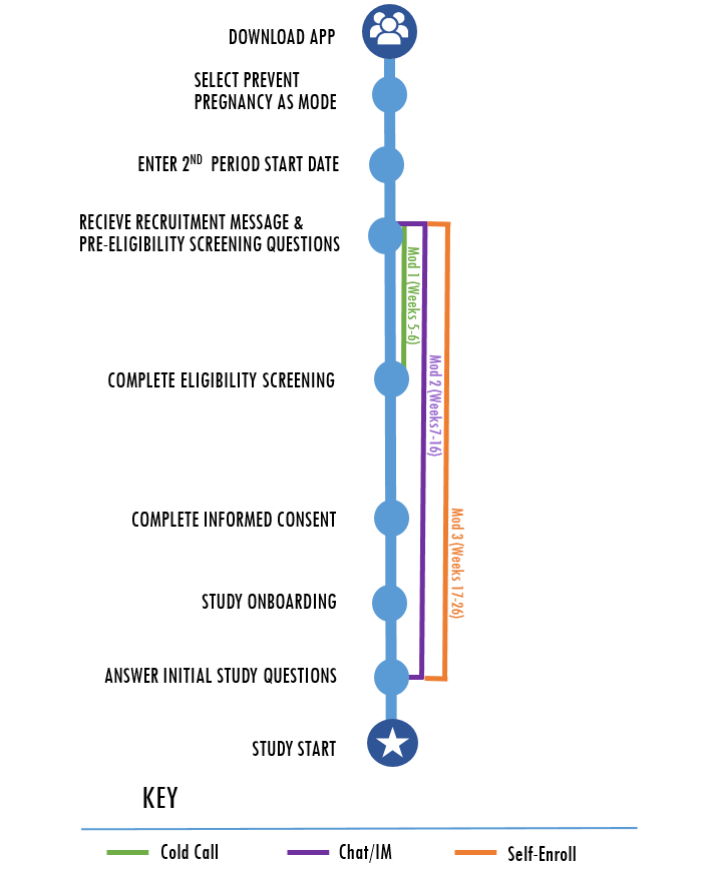
Recruitment process with modification impact zones.

**Figure 3:**
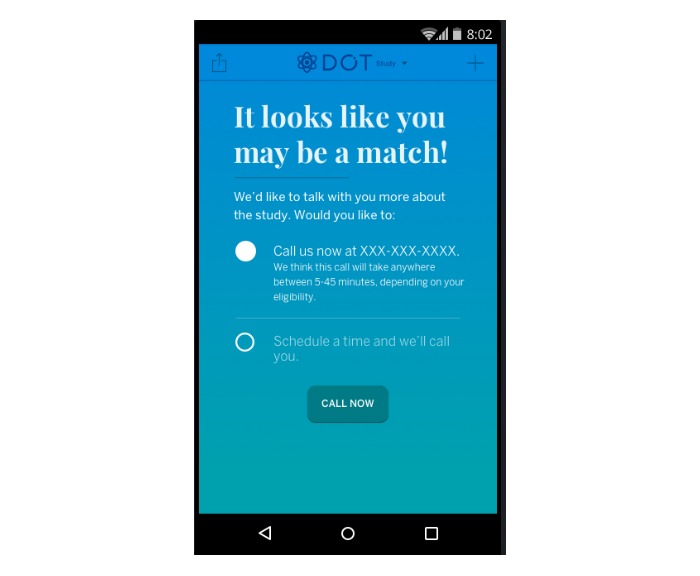
The pop-up screen after women were determined to be pre-eligible.

To receive the recruitment message to participate in our study, there were 2 requirements. As depicted in Figure 2, the women had already downloaded the Dot app onto their Android phones, and second, they had designated their intention to prevent pregnancy [10] (rather than to get pregnant or track their cycles). Once users in this pool entered their second period start date, a pop-up message describing the study appeared on their app asking whether they were interested in participating in the study. Sending this message immediately after a woman entered her second period start date ensured that she was not pregnant on entry to the study [10]. It also increased the likelihood that potential participants would be actual app users rather than someone who downloaded the (free) app out of curiosity and did not actually use it. Women who were interested in participating in the study received prescreening pop-up questions about their age (18-39 years), fertility intention (prevent pregnancy), and recent use of hormonal contraception [10]. Pre-eligible women were then (1) further screened for study eligibility, (2) provided with more information about the study, (3) led through an informed consent process, and (4) enrolled in the study [10]. To minimize the risk of pregnancy before enrollment, women were required to complete this process within 6 days [10] of entering their period start date. Figure 3 reflects the pop-up screen women received on their smartphone after being identified as pre-eligible for the study.

### Original Approach

In the original strategy, women who had responded appropriately to the prescreening questions spoke on the phone to study representatives who led them through the full enrollment questionnaire as well as the informed consent document. This informed consent process, which was approved by the Georgetown University’s IRB, required that the participants initial the document in their app and verbally consent to a study representative that they agreed to participate. On agreement, the study representative was able to activate Proofmode on the participant’s phone. Study representatives then conducted a brief onboarding process, explaining Proofmode and the data it collects to ensure that participants were familiar with and agreed to what the study was asking of them. Participants followed this process with visual onboarding screens on their phones and were given the opportunity to ask questions about the study and the features of the research interface. Once onboarding was complete, study representatives then administered a brief sociodemographic survey that included information about age, ethnicity/race, and education, among others.

**Figure 4:**
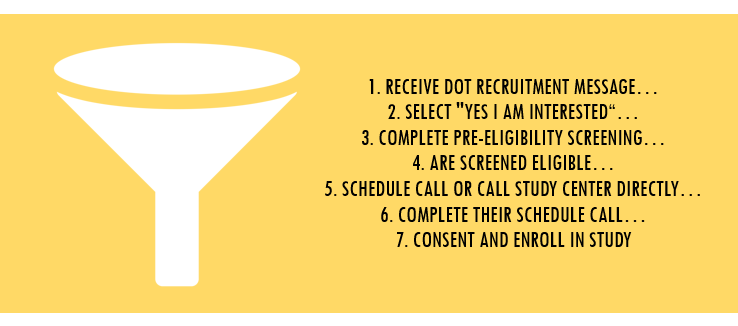
Dot study recruitment and enrollment process funnel.

Within the first month of the 6-month recruitment and enrollment period, only a small number of users who initially indicated interest in the study actually enrolled (described in detail in the following). Due to the low recruitment rates using this method, the recruitment procedures were reconsidered. Tailoring the recruitment “funnel,” or the way users are guided to the goal with fewer navigation options at each step, began with an assessment of the app’s user analytics data with our technology partner (EastBanc Technologies) and the app developer (Cycle Technologies) to identify “leakage” places within the recruitment funnel where potential participants do not continue to full enrollment or how we were losing potentially interested participants at each step of the process (Figure 4).

The changes described in the modifications below reflect a series of meetings and decision points that our team implemented in conjunction with EastBancTechnologies and Cycle Technologies. In collaboration, we identified problems within recruitment process and brainstormed solutions. We also developed long- and short-term contingency plans that included benchmarks for recruitment numbers. Finally, solutions were pilot-tested by the teams to ensure seamless implementation. On any new updates to the app or Proofmode, participants received push notifications from the study team.

### Strategy: Establish Recruitment Leakage Points

To identify the causes of leakage and adapt the process accordingly, we used aggregate data from the Google Play store, app user analytics data using Amplitude analytic software, and data from Proofmode to review the funnel.

### Understanding the Funnel

Figure 4 describes the funnel, which comprised a series of 7 steps from the time a woman selected “Yes, I’m Interested” in response to the recruitment message to the time she was enrolled in the study.

Over the first 4 weeks, 690 users received the recruitment message, 176 were interested in participating in the study, of whom 103 (103/176, 62.1%) were eligible; but only 22 eligible participants completed enrollment in the study (22/103, 21%). This conversion rate was far lower than what we needed to achieve our enrollment goal (a minimum of 700 women) in the time available. We determined that reducing leakage at this point would require increasing the likelihood of scheduled or immediate calls.

#### Modification 1: First Point of Study Contact (Weeks 5-6)

In the original recruitment process, lack of completion of scheduled calls with study representatives appeared to be the first point of leakage. Thus, the main change in modification 1 was the integration of a call reminder. We presumed that increased awareness might decrease the likelihood of women rejecting or forgetting about the call.

The following steps were taken to reduce leakage at this point in the process:

- Increased visibility of the study center contact information, which was pinned to the app home screen for women who reported interest but did not enroll. The contact screen required the women to either exit the reminder screen or select a contact option.
- Created a protocol for identifying eligible women who scheduled a call and provided appointment reminders for those scheduled calls.
- Established a phone contact protocol to reach for eligible women who indicated interest and were considered pre-eligible after completing the pre-eligibility questions but had not yet called or scheduled a call. Study representatives called the identified women. If the woman did not answer, representatives left a voicemail explaining the limited window of time for enrollment and providing contact information.
- Integrated a call scheduling feature into the app that allowed women to schedule a call only until the last day of their enrollment window.
- Created appointment syncing functionality with Google calendars.

After modification 1, a total of 1907 users received the recruitment message, 460 identified interest in the study, and 267 were eligible. Still, only 60 of the eligible participants enrolled in the study (60/267, 22.5% success).

#### Modification 2: Enabling a Research Chat/Instant Message Feature (Weeks 7-15)

We learned from the first modification that it was acceptable to call eligible women proactively, but many women allowed these calls to go to voicemail. When contact was made, conversations reflected 2 main themes: first, women had additional questions about what was required of them in the study; second, women had assumed they were already enrolled after completing the pre-eligibility questions. In the second modification, we instituted a “Chat with us now” feature by integrating Intercom, a software that incorporated chat functionality into Proofmode (Figure 1). This facilitated multiple simultaneous chat conversations between users and study representatives, as shown in Textbox 1. This feature was widely used, and the team found that women were comfortable communicating through the chat feature.

**An example of a potential participant using the “Chat with us now” feature to ask a question.**

Hi! I’m interested in being part of the Dot study. It seems like a great technological advancement for women’s health, so I don’t mind contributing my data. Will my information be collected automatically just by using the app?

Potential participant on chat

**Reminder messages about recruitment were sent at different points through different mechanisms in Intercom.**

Hi there, this is the Dot Study team. Time’s running out to enroll in our research study- we only recruit for six days after your cycle starts. If you think you might be interested, feel free to reach out to us. We’re available via chat, or you can always call our study center.

Scripted 48 hour “Reminder” Text

Hi there! Today is the last day for you to enroll in the Dot study via chat or call us before your time expires! We’re happy to answer any questions you might have. Our hours are Monday- Friday 9am – 5pm EST. You are always welcome to contact our study center at XXX-XXX-XXXX to enroll as well. Let us know!

Scripted 24 hour “Reminder” Text

Dot Study Representative

Textbox 2 shows a series of standardized messages that were sent to participants to facilitate conversations and encourage enrollment. All participant conversations were archived for future analyses. The chat feature provided a user-friendly tool to communicate the enrollment process to participants and also was linked with the database, providing study staff with the ability to enroll participants directly. Textbox 1 provides exemplary chat interactions with participants.

In addition, standardized messages based on user behaviors were tested by our technology partner to identify which types of communication (in-app messages, push messages, automated messages, or manual messages to specific participants) were most effective at eliciting a response. After identifying the most effective mechanism for communication, study protocols were updated. We also learned that many women were not aware that the enrollment window was limited to 6 days. Addressing this challenge, an automated enrollment message was sent through Proofmode to remind potential participants of their enrollment deadline, both at 48 and 24 hour before their enrollment window ended.

The changes integrated into this modification are summarized in the following:

- Integrated Intercom chat software into Proofmode, which enabled chat use for women who responded with interest in the study.
- Included a “Chat with us now” option within the research interface dropdown menu. Women who reported interest in the study received a message, allowing them to engage in a chat session with study staff at their convenience.
- Identified enrollment windows and implemented standardized push reminders informing women when their enrollment window was closing.

After modification 2, a total of 5089 users received the recruitment message, 1067 identified interest in the study, and 674 were eligible. However, only 143 eligible participants were converted to study enrollment (143/674, 21.2%).

#### Modification 3: Self-Enroll Mechanism (Recruitment Weeks 16-26)

After examining the rate of enrollment after the integration of chat functionality and considering feedback from current participants, we decided to integrate a fully automated enrollment functionality into Proofmode. On approval from the IRB, we implemented an option that enabled participants to complete both the full eligibility screening and the informed consent process solely through the app. Participants were also provided complete access to the chat or phone call options that were integrated earlier.

The major consideration for the study team was the transition from an informed consent process that was facilitated through live interaction between study representatives and potential participants to a fully automated consent process. There are numerous examples of app-based (automated) informed consent procedures in mHealth research [11,12]. In several studies, electronic signatures were identified as sufficient to validate the consent process [13,14]. Women in the Dot study had 3 choices: (1) completing the informed consent through a fully automated process, (2) having a phone call with study representatives, or (3) using chat functionality. Again, study protocols and automated response messages were generated and tested by the study team before implementation. Regardless of which option the women chose, each provided the opportunity to communicate with a live person to ask any questions or resolve any concerns before signing the informed consent document.

During the 10 weeks that modification 3 was implemented, most women used self-enroll (366/494, 74.1%), followed by chat (90/494, 18.2%), and phone (38/494, 7.7%). After modification 3, a total of 6451 users received the recruitment message, 1311 identified interest in the study, 715 were eligible, and 494 eligible participants were converted to study enrollment (494/715, 69.1%).

## Results

### Recruitment and Enrollment

Data from the Amplitude user interface portal were analyzed to show how each modification impacted the number of women actually enrolling in the study. During the enrollment period, 719 women enrolled in the study. Weekly enrollment increased after the first modification, then decreased until the final modification was implemented, at which point the overall enrollment numbers went up significantly and conversion rates improved (Table 1).

**Table 1:** Percentage change in enrollment by modification.

Recruitment strategies and modifications	Weeks implemented (total weeks)	Number enrolled	Mean participants per week	Percentage change in weekly enrollment
Original recruitment strategy	1-4 (4)	22	5.5	—^a^
Modification 1	5-6 (2)	60	30	500
Modification 2	7-15 (8)	143	17.9	40.3
Modification 3	16-26 (10)	494	49.4	176
Total		719		

^a^There was no change in recruitment during the first phase as this was the original recruitment strategy.

**Table 2:** Recruitment and enrollment funnel results during each modification strategy. N/A: not applicable.

Key funnel indicators	Original strategy	Modification 1	Modification 2	Modification 3
Time frame	Weeks 1-4	Weeks 5-6	Weeks 7-15	Weeks 16-26
Number of downloads	27,364	19,801	28,478	54,018
Estimated women preventing pregnancy with Dot^a^	9030	6534	9398	17,826
Received recruitment message	690	1907	5089	6451
Indicated interest in the study, n (%)	176 (25.5)	460 (24.12)	1067 (20.97)	1311 (20.32)
Completed pre-eligibility screening questions, n (%)	166 (94.3)	448 (97.4)	1038 (97.28)	1280 (97.64)
Eligible for the study and given enrollment options, n (%)	103 (62.1)	267 (59.6)	674 (64.93)	715 (55.86)
Scheduled call confirmation screen, n (%)	62 (60.2)	149 (55.8)	29 (9.8)	8 (1.1)
Called immediately, n (%)	4 (4)	14 (5.2)	4 (0.5)	25 (3.5)
Enrolled total	22	60	143	494
Conversion rate (%)^b^	21.4	22.5	21.2	69.1
Enrolled via chat, n (%)	N/A	N/A	53 (37.4)	90 (18.2)
Enrolled via self-enroll, n (%)	N/A	N/A	N/A	366 (74.1)

^a^Information on the proportion of users using Dot to prevent pregnancy obtained from Cycle Technology suggests that 33% of Dot users are preventing pregnancy.

^b^Conversion rate calculated by dividing the number of enrolled women by the number of women identified as eligible for the study and given enrollment options.

Through all of these changes, there were certain steps in the recruitment funnel that remained constant. The percentage of women who declined the recruitment message or chose “Ask me later” did not change significantly. This is also true of the percentage of women who answered the pre-eligibility questions and were screened ineligible.

Table 2 shows the flow of participants through the recruitment and enrollment process from the original strategy through modification 3.

### Demographics

Chi-square comparisons of participant demographics are presented in Table 3. We found that as we implemented the modification, the percentage of women in each age category shifted slightly and not significantly across the modifications (χ^2^_9_=16.3, *P*=.06). Individuals aged 18 to 29 years comprised about two-thirds (440/719, 1.2%) of participants throughout enrollment process, whereas 30- to 34-year-olds made up a little more than one-fifth of the participant base (158/719, 22.0%).

Participants’ race or ethnicity changed descriptively across the modification as well, but with limited impact (χ^2^_21_=30.5, *P*=.08) on the generalizability of study findings. The percentage of white participants remained relatively high (391/719, 54.4%) throughout recruitment, whereas black and Hispanic enrollment shifted with each change in process and comprised 18.3% (132/719) and 15.7% (113/719) of our overall participant base, respectively. We found the highest percentage of black and Hispanic participants enrolling after modifications 3 and 4.

Comparisons of participants’ education level across each enrollment modification reflected significant differences (χ^2^_15_=36.5, *P*<.001). Of note, a higher percentage of enrolled women reported their education as “some college” after each modification, whereas the inverse was true for women reporting that they completed their “bachelor’s degree” (Table 3).

**Table 3:** Demographic distribution of participants during the enrollment modification.

Demographic characteristics		Original (N=22)	Modification 1 (N=60)	Modification 2 (N=143)	Modification 3 (N=494)
		n (%)	n (%)	n (%)	n (%)
**Age^a^**					
	Verified 18-39	0 (0)	5 (8)	7 (4.9)	3 (0.6)
	18-24	2 (9)	20 (33)	45 (31.5)	147 (29.8)
	25-29	9 (41)	17 (28)	50 (35.0)	150 (30.4)
	30-34	9 (41)	13 (22)	28 (19.6)	108 (21.9)
	35-39	2 (9)	5 (8)	13 (9.1)	86 (17.4)
**Race/ethnicity^b^**					
	No response	1 (5)	6 (10)	8 (5.6)	20 (4.0)
	Black/African American	6 (27)	8 (13)	31 (21.7)	87 (17.6)
	Hispanic or Latino	2 (9)	9 (15)	15 (10.5)	87 (17.6)
	White	12 (55)	31 (52)	79 (55.2)	269 (54.5)
	Other	1 (5)	6 (10)	10 (7.0)	31 (6.3)
**Education level^c^**					
	No response	0 (0)	4 (7)	11 (7.7)	20 (4.0)
	High school/ GED	3 (14)	14 (23)	16 (11.2)	83 (16.8)
	Trade/vocational school	1 (5)	1 (2)	3 (2.1)	41 (8.3)
	Some college	8 (36)	20 (33)	64 (44.8)	226 (45.7)
	Bachelor's degree	9 (41)	16 (27)	30 (21.0)	86 (17.4)
	Postgraduate degree	1 (5)	4 (7)	19 (13.3)	38 (8.0)

^a^Note that participants are not required to give exact age but simply confirm to be between 18 and 39 years old.

^b^Note that responses to race/ethnicity and education level are not mandatory.

^c^*P*<.05.

The percentage of participants who reported completing some college increased across the modifications, whereas those reporting a bachelor’s degree decreased over time (Table 3). The percentage of participants with a high school diploma or General Equivalency Development was highest after modification 1 but dropped under 20% in subsequent modifications. Also, with each modification, the percentage of participants with a college degree decreased.

## Discussion

### Principal Findings

Our intention was to recruit into the Dot study women who understood that they were participating in a study and what study participation involved, recognized the importance of consistent data entry, and had the potential to complete up to 13 cycles of use. At the same time, we wanted to minimize participants’ interaction with study staff because this might have an effect on study results. In addition, we wanted our study population to reflect the general population of the United States to maximize generalizability of results. Our experience suggests that it is possible to achieve this balance, but that app-based research requires agile and creative approaches that increase clarity and communication with potential participants during the recruitment-to-enrollment process. Findings reveal that a more automated and self-guided enrollment process was preferred by many of the Dot study participants. The percentage of women who converted to participant status after modification 3 was 69.1% (494/715) versus a conversion rate of approximately 21% for the original and 2 earlier modifications.

Each modification reflected varying levels of contact and interaction with women through the enrollment process and required different behaviors on the part of potential study participants. Modification 1 targeted women who were eligible for the study, whether they did or did not schedule a call, by having study representatives remind them of their possible eligibility and/or scheduling phone calls. This tripled our enrollment, yet the trajectory for achieving enrollment requirements was still not within the requisite enrollment time frame. This led us to implement modification 2, which gave women the alternative of interacting with study staff, asking questions, and enrolling in the study via chat. The value of the chat functionality was apparent quickly. As a result, we maintained this feature throughout enrollment and the study to provide women with the opportunity to continue asking questions through the study. Chat engagement was managed by the study team and used a series of prewritten responses and protocols. The implementation of modification 3 resulted in a 176% increase in weekly enrollment. Although almost 75% of the participants self-enrolled during this modification, it was beneficial to maintain phone calls and chat functionality throughout the process. We received positive feedback from participants about the chat feature in particular; although many chose to self-enroll, participants liked the fact that they could easily ask questions through chat about the study and the enrollment process.

### Key Learnings and Participant Characteristics

Studies have investigated the role of the recruitment process as it pertains to “contact timing, content of the subject line, and incentives” [15-17], but we were unable to find an example that recruited in a similar manner as in this study. A systematic review of studies using Facebook for recruitment found that participants were often compared with a control arm or another study arm using traditional methods [18]. They also found that the demographics of participants recruited through Facebook were “relatively representative,” but the authors cited several exceptions often based on study criteria [18]. Although Facebook advertisement was used to promote the Dot app, participants were recruited through the existing pool of Dot users who entered their second period start date. Anecdotally, participants reported seeing ads for Dot on a range of sources that included news articles, Instagram, and the Google Play Store, but more than two-thirds traced back to Facebook advertisements. Thus, our sample predominantly reflects women who are both Facebook users and women who were interested in downloading the Dot app. Unfortunately, systems do not facilitate comparing the demographics of women who downloaded Dot and chose not to enroll with those who did.

In this study, advertisement through Facebook and the enrollment processes resulted in a diverse sample, but variation in participant demographics was descriptively different across the modifications in the enrollment process. This variation may merit further investigation on the influence of internet/app-based enrollment procedures on participant inclusion. Such analyses are beyond the scope of these data and would require more intentional variation in enrollment procedures. For the purposes of this study, poststudy analyses will describe retention of participants across a number of variables, including enrollment procedure to provide guidance for future studies.

Comfort and familiarity with mobile technologies and digital apps may vary across the participant pool. As presented previously, self-enrollment was consistently near or above 50% among all age groups, whereas one-fourth to about one-fifth used the chat feature. The decrease in call frequency during modifications 2 and 3 may have been due to participants’ ease and familiarity with IM/chat and self-enroll procedures for other purposes. Given that a significantly higher proportion of women were enrolled through the self-enrollment mechanism, it can be assumed that participants preferred to engage with the study without the aid of study representatives, regardless of age category. Although there were some differences in demographic characteristics of women enrolling across the 3 modifications, the diversity of the study population is reassuring and suggests that study results will be generalizable in the United States and potentially in other settings as well.

### Conclusions

Studies have shown that mHealth apps have the potential to address a number of needs across several health areas [19,20], but recruiting and enrolling participants into prospective mHealth studies to understand apps’ effectiveness is challenging. The Dot efficacy study represents an attempt to apply criteria for high-quality research, in this case, a contraceptive efficacy study, to a technology that is largely unstudied.

Each step in the study process thus presents unknown challenges with few guidelines for establishing best practices for success. With the rapid expansion of the availability and use of personal information apps, it is critical that we understand their implications for health outcomes. Rigorous studies, similar to those focused on more traditional approaches to providing information, are needed. Our experience provides several suggestions for recruiting and enrolling adequate number of participants to app-based studies. Broadly, we encourage future app researchers to be agile in their approach to recruitment, reviewing, and responding quickly to issues as they arise, while maintaining both ethical standards and rigorous research. Recommendations based on our experiences include the following:

1. Establish an ongoing relationship with developers and technology partners to quickly identify and resolve issues as they arise.
2. Before recruitment launch establish minimum recruitment numbers by certain dates and identify potential pivot strategies based on various outcome scenarios, as well as their potential budget impacts.
3. Invest in and use analytic and monitoring data to provide real-time insights to successes and challenges to use true data to decision making.
4. Foster a “fail fast fail forward” mindset among all partners from the beginning, so that everyone will be on board with implementing strategic shifts in a timely manner.
5. Budget for likely changes to strategy and approach.

With this picture of the Dot efficacy study population, we now intend to continue monitoring participants through Proofmode and Amplitude to observe whether women who enrolled through different approaches and with different sociodemographic characteristics perform differently vis-à-vis their daily sexual behavior data (ie, whether they enter data as frequently; whether during the days Dot identifies as “risk” days for pregnancy, they have unprotected intercourse or use condoms, withdrawal, and/or emergency contraception as frequently). We will also analyze quantitative and qualitative data on partner communication and supportiveness, app perception, and fertility awareness knowledge collected through periodic surveys.

Implementing changes in the recruitment-to-enrollment process required both technical adjustments to Proofmode and protocol amendments to our IRB and thus took time. Future studies that wish to implement agile recruitment strategies should include contingency recruitment strategies that factor in the time for changes to occur. In addition, recruitment planning should predesignate benchmarks to assess recruitment and enrollment success and ensure that ineffective processes are quickly identified and addressed.

## Abbreviations

Dot: Dynamic Optimal Timing
IRB: Institutional Review Board
IRH: Institute for Reproductive Health
IM: instant message
